# Rapid isothermal molecular tests to discriminate between *Leishmania braziliensis* and *Leishmania infantum* infections in dogs

**DOI:** 10.1186/s13071-024-06633-7

**Published:** 2025-01-07

**Authors:** Rafaela Lira Nogueira de Luna, Kamila Gaudêncio da Silva Sales, Lucas Lisboa Nunes Bonifácio, Luciana Aguiar Figueredo, Thomas R. Shelite, Fábio dos Santos Nogueira, Domenico Otranto, Filipe Dantas-Torres

**Affiliations:** 1 Aggeu Magalhães Institute, Oswaldo Cruz Foundation (Fiocruz), Recife, Brazil; 2 Department of Biosafety, University of Texas Medical Branch, Galveston, USA; 3 Andradina Educational Foundation, Andradina, Brazil; 4 Department of Veterinary Medicine, University of Bari, Valenzano, Bari, Italy; 5 Department of Veterinary Clinical Sciences, City University of Hong Kong, Hong Kong, China

**Keywords:** Isothermal amplification, Leishmaniasis, Molecular diagnosis, Point-of-care, Recombinase

## Abstract

**Background:**

We standardized two recombinase polymerase amplification (RPA) assays coupled with lateral flow (LF) strips for the detection of *Leishmania braziliensis* and *Leishmania infantum* kinetoplast DNA (kDNA).

**Methods:**

The RPA-LF assays were tested at different temperatures and reaction times, using DNA from cultured *L. braziliensis* and *L. infantum*. The *L. infantum* RPA-LF was also tested using clinical samples (bone marrow and skin) from infected and uninfected dogs.

**Results:**

The detection limits (analytical sensitivity) of the assays were 0.04 pg/μl and 0.04 ng/μl for *L. braziliensis* and *L. infantum* kDNA, respectively. Using clinical samples, the *L. infantum* RPA-LF successfully detected the parasite kDNA in bone marrow (21/30; 70.0%) and skin samples (23/30, 76.6%) from naturally infected dogs. We found an almost perfect agreement (kappa = 0.807) between RPA-LF for *L. infantum* and our reference quantitative real-time polymerase chain reaction (qPCR), considering clinical samples with a quantification cycle (*C*_q_) < 30, whereas the agreement with samples with a *C*_q_ > 30 (lower parasite loads) was moderate (kappa = 0.440).

**Conclusions:**

The RPA-LF assays developed here may be promising diagnostic tools for point-of-care diagnosis of *L. infantum* and *L. braziliensis* infection in dogs, particularly in remote rural areas lacking laboratory infrastructure.

**Graphical Abstract:**

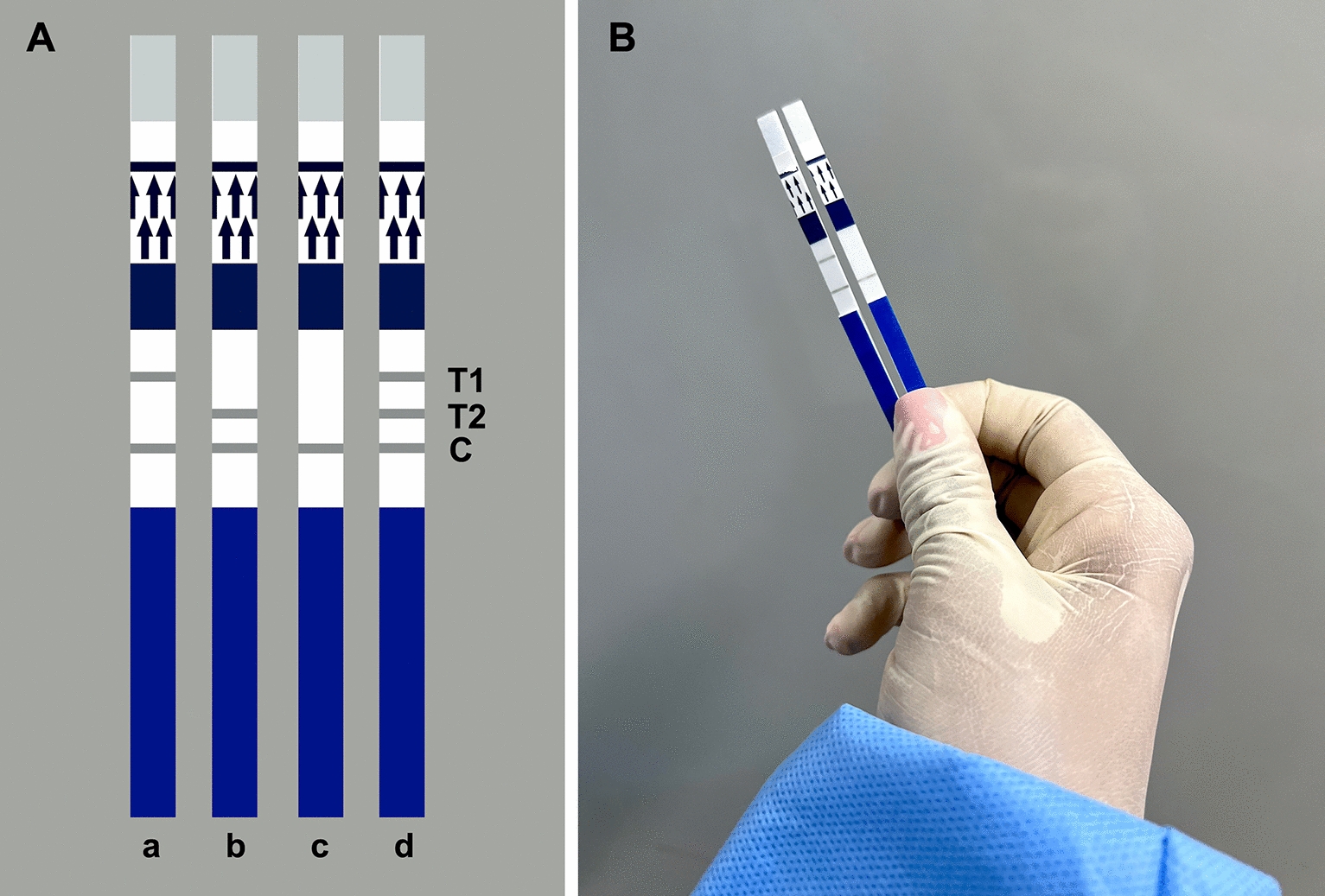

## Background

Canine leishmaniasis is a parasitic disease caused by various species of protozoa belonging to the genus *Leishmania* [1, 2]. In the American continent, *Leishmania braziliensis* and *Leishmania infantum* are among the most common and widespread agents of canine leishmaniasis [3]. In areas where different *Leishmania* spp. co-exist, co-infections may occur and pose an additional challenge for the etiological diagnosis of leishmaniasis in dogs [4–6].

From a clinical perspective, the disease produced by various *Leishmania* spp. in dogs may vary widely [3, 7], with localized ulcers being the main clinical hallmark of the disease caused by *L. braziliensis* [8, 9], sometimes associated to secondary mucocutaneous lesions [10], just as in humans [11, 12]. However, *L. infantum* may also cause a wide range of clinical signs and clinicopathological abnormalities, varying from mild skin lesions to systemic signs, being potentially life-threatening [3, 7]. Despite the apparently distinct clinical expression of the diseases caused by *L. braziliensis* and *L. infantum*, the diagnosis of canine leishmaniasis in areas where these agents co-occur remains challenging. Indeed, dogs infected by *L. braziliensis* are often from remote rural areas, where the access to veterinary healthcare is limited and malnutrition associated to other conditions is common [13, 14]. Consequently, *L. braziliensis*-infected dogs may present clinical signs and clinicopathological abnormalities (e.g., weight loss, anemia, and thrombocytopenia) [8] that are not primarily caused by *L. braziliensis* but may contribute to the general clinical picture, thus further confounding the diagnosis.

To complicate matters, serological assays are widely used as diagnostic tools, by both veterinary practitioners and public health workers. Although these assays may present high sensitivity and specificity, they are prone to cross-reactivity, and the consequences may be severe [5, 15–19]. For instance, a study conducted in Rio de Janeiro, Brazil, evaluated whether dogs euthanized based on serological results were, in fact, infected by *L. infantum* [5]. This study proved that many culled dogs were infected by *L. braziliensis* or even *Trypanosoma caninum* (unknown pathogenicity), highlighting that serological cross-reactivity resulted in the unnecessary death of dogs that were, in fact, not infected by *L. infantum*. This exemplifies the importance of using molecular tools that can properly identify the species of *Leishmania*, before deciding the fate of an infected dog.

The search for point-of-care molecular tests has intensified in recent years, with promising results [20, 21]. One of the alternative methods that has been explored in the past for the point-of-care detection of *Leishmania* DNA is recombinase polymerase amplification (RPA) [20, 22, 23]. Originally developed in 2006, RPA is an isothermal amplification using proteins involved in cellular DNA synthesis, recombination, and repair [24, 25]. RPA does not require an initial denaturation step to generate single-stranded DNA from target double-stranded DNA, in contrast to polymerase chain reaction (PCR)-based molecular techniques, which highlights its suitability for field use [26, 27]. Various studies have investigated the use of RPA-based assays for the detection of *Leishmania* spp., with encouraging results in terms of sensitivity and specificity [20, 22, 23].

In this study, we standardized two new RPA assays coupled with lateral flow (LF) reading for detecting *L. braziliensis* and *L. infantum* in skin and bone marrow samples from naturally infected dogs in Brazil.

## Methods

### Strains and clinical samples

Reference strains of cultured *L. braziliensis* (MHOM/BR/1975/M2903) and *L. infantum* (MHOM/BR/1974/PP75) were used to obtain standard DNA. Clinical samples (*n* = 70) from dogs (34 skin and 36 bone marrow samples) were also used to validate the standardized RPA-LF assays. These samples were from a previous study conducted in Andradina, São Paulo, and were originally categorized as negative (*n* = 10) and positive (*n* = 60) based on results obtained by quantitative real-time PCR (qPCR) (unpublished data).

### DNA extraction

Extraction of genomic DNA from *Leishmania* spp. strains and clinical samples from dogs were performed using the DNeasy Blood & Tissue Kit (Qiagen, Germantown, MD, USA) following the manufacturer’s instructions. The quantity and purity (absorbance ratios at 260/280 nm and 260/230 nm, respectively) of the DNA extracted from the strains were evaluated using a spectrophotometer (NanoDrop Lite, Thermo Scientific, Waltham, MA, USA). The extracted genomic DNA was frozen at –20 °C until use.

### qPCR and conventional PCR (cPCR)

The fast qPCR assay originally used to categorize samples as positive or negative targeted a 120-base-pair (bp) fragment of the kDNA minicircle of *L. infantum*, employing the primers LEISH-1 (5′-AACTTTTCTGGTCCTCCGGGTAG-3′) and LEISH-2 (5′-ACCCCCAGTTTCCCGCC-3′) and the TaqMan^®^ probe FAM-5′-AAAAATGGGTGCAGAAAT-3′ non-fluorescent quencher minor groove binder (MGB) [28]. The reagent concentrations and thermal cycling conditions were as described elsewhere [29]. All samples were tested in duplicate.

A cPCR assay, employing the same primers as for qPCR, was used for comparison purposes, with the following thermal cycling conditions: 94 °C for 3 min, followed by 35 cycles at 94 °C for 30 s, 58 °C for 30 s, and 72 °C for 30 s; with a final extension at 72 °C for 5 min. The cPCR was conducted in a 25 µl final reaction mixture containing 12.5 μl of GoTaq^®^ Colorless Master Mix (Promega Corporation, Madison, WI, USA), 2.25 μl of each primer (900 nM final concentration), 6.0 μl of nuclease free water, and 2.0 µl of DNA template.

### Primers and probes used in the RPA assays

Primers and probes used in the RPA for detecting *L. braziliensis* and *L. infantum* kinetoplast DNA (kDNA) were those described by Saldarriaga et al. [23] and Castellanos-Gonzalez et al. [22], respectively. These primers target a 120-bp and a 182-bp region of the kDNA minicircle of *L. braziliensis* and *L. infantum*, respectively. To enable detection of amplicons using LF strips, primers and probes were modified according to recommendations contained in the TwistAmp^®^ Assay Design Manual (TwistDx, Cambridge, UK). The probe for each target was differentially labeled at the 5′ end with carboxyfluorescein (FAM) and digoxigenin (DIG) for detection of *L. braziliensis* and *L. infantum*, respectively. Additionally, an internal abasic nucleotide analogue (dSpacer) and a polymerase extension blocking group at the 3′ end (spacer C3) were added to the probes. The reverse primers for each target were also chemically modified with a biotin at the 5′ end. Primers and probes were synthesized by LGC Biosearch Technologies (Middlesex, UK). The sequences of the primers and probes used herein are shown in Table 1.

**Table 1:** Primers and probes used in the recombinase polymerase amplification (RPA) assays

Primer/probe	Sequence (5′ → 3′)	Amplicon size	Reference
LB-F	GATGAAAATGTACTCCCCGACATGCCTCTG	120 bp	[23]
LB-R	Biotin-CTAATTGTGCACGGGGAGGCCAAAAATAGCGA		
LB-P	FAM-GTAGGGGNGTTCTGCGAAAACCGAAAAATG-dSpacer-CATACAGAAACCCCG-Spacer C3		
LI-F	CCATAGCGCTTTAGAATAGTTCGACTCCGA	182 bp	[22]
LI-R	Biotin-ATCGGTATAGATATTACTACTACACACAGC		
LI-P	T(DIG)-ATAACTGACATTACTCGTACACTATAA-dSpacer-TATTATGTTTAATATAT-Spacer C3		

*LB-F*, *L. braziliensis* forward primer; *LB-R*, *L. braziliensis* reverse primer; *LB-P*, *L. braziliensis* probe; *LI-F*, *L. infantum* forward primer; *LI-R*, *L. infantum* reverse primer; *LI-P*, *L. infantum* probe

### Analytical sensitivity and specificity

Standard curves were prepared with five serial dilutions (5 ng/µl, 0.5 ng/µl, 50 pg/µl, 5 pg/µl, and 0.5 pg/µl) of DNA extracted from *L. braziliensis* and *L. infantum* reference strains. Standard curves were used to evaluate the analytical sensitivity (i.e., limit of detection) of the RPA assays. In turn, the analytical specificity of the RPA assays was evaluated using cross experiments, that is, adding *L. braziliensis* DNA in the RPA targeting *L. infantum* and vice versa. All experiments included a no-template control (i.e., all the reagents, except the DNA template).

### RPA reaction and LF reading

RPA reactions were performed using the commercial TwistAmp Basic kit (TwistDx, Cambridge, UK). The protocol suggested by the manufacturer was adapted to enable the visualization of amplicons on the LF strips. For this purpose, we added the reaction mixture, exogenous NFo (Intact Genomics, St. Louis, MO, USA), which typically provides the generation of a double-labeled product, which can then be detected in the LF strips. Furthermore, during the standardization process, other modifications in the concentration of reagents, temperature, and reaction time were made to the standard protocol to optimize the assays.

**Fig. 1:**
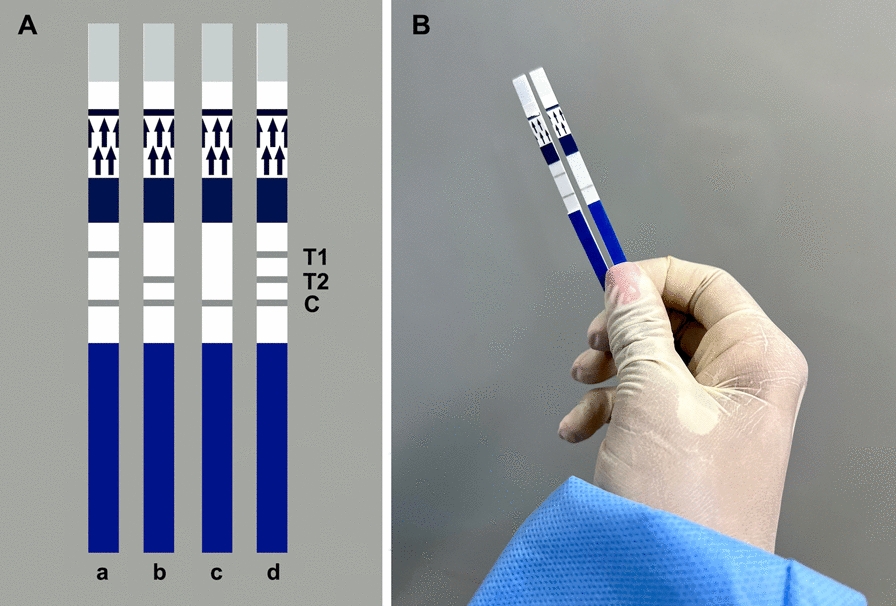
Schematic illustrations of the PCRD FLEX lateral flow (LF) strips (Abingdon Health, York, UK) (**A**): **a**, positive result for test line 1 (T1); **b**, positive result for test line (T2); **c**, negative result showing control line (C); **d**, an illustration of all lines (T1 detects DIG/biotin-labeled amplicons; T2 detects FAM/biotin- or FICT/biotin-labeled amplicons; C is the control line). Actual examples of a T1-positive (left) and a T1-negative (right) result with the recombinase polymerase amplification-LF assay (RPA-LF) for *L. infantum* (**B**)

To select the optimal incubation conditions, the RPA assays were performed at different temperatures (20, 25, 30, 35, 40, 45 and 50 °C) in a previously established amplification time (40 min). Also, the tests were carried out at different time periods (5, 10, 15, 20, 25, 30, 35 and 40 min). RPA assays were performed in a dry bath incubator (DB – Heat & Cool, Loccus, São Paulo, SP, Brazil). Immediately after isothermal amplification, the amplicons were visualized using PCRD FLEX LF strips (Abingdon Health, York, UK), following the manufacturer’s instructions (Abingdon Health, York, UK). PCRD FLEX LF strips detect up to two differently labeled amplicons. Thus, test line 1 (T1) detects DIG/biotin-labeled amplicons, whereas test line 2 (T2) detects FAM/biotin-labeled amplicons (Fig. 1). Test line 3 (T3) is the control line.

### Data analysis

The sensitivity, specificity, accuracy, and positive and negative predictive values for the *L. infantum* RPA-LF assay were calculated as described elsewhere [30], using either qPCR or cPCR as a reference test. The strength of agreement between the PCR assays (both qPCR and cPCR) and RPA-LF assay for *L. infantum* was assessed using kappa statistics and categorized as follows: slight (0–0.20), fair (0.21–0.40), moderate (0.41–0.60), substantial (0.61–0.80), and almost perfect agreement (0.81–1.00) [31]. Data were analyzed using QuickCalcs of GraphPad Software (https://www.graphpad.com/quickcalcs/).

## Results

### Standardization of RPA-LF assays

**Fig. 2:**
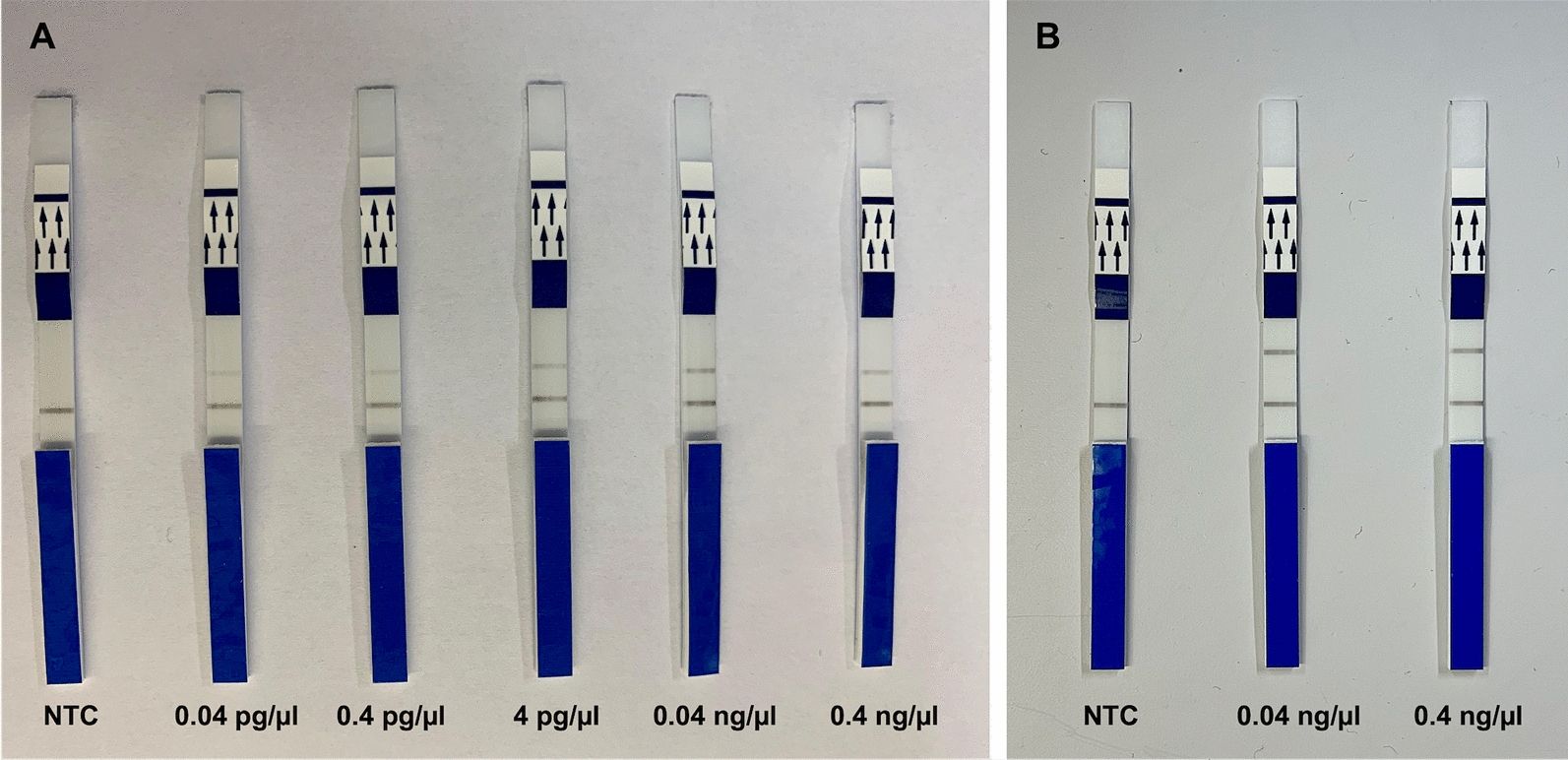
Representative examples of the recombinase polymerase amplification–lateral flow assays (RPA-LF) for *L. braziliensis* (**A**) and *L. infantum* (**B**). Standard DNA was obtained from cultured promastigotes of reference strains (see Methods for details), and a master mix without DNA template was used as no-template control (NTC). The assays for *L. braziliensis* and *L. infantum* were performed at 45 °C and 40 °C, respectively, for 40 min

The concentrations of the reagents of the RPA-LF assays for detecting *L. braziliensis* and *L. infantum* are described in Tables 2 and 3. These concentrations were established adjusting the concentrations suggested by the manufacturer of the TwistDx master mix, based on results obtained in terms of analytical sensitivity. In the RPA-LF assay for *L. braziliensis*, the primers and probe were used at a concentration of 5 µM (Table 2), allowing the detection of all DNA concentrations used in the standard curve. In terms of incubation temperature and time, the initial conditions tested, that is, 45 °C for 40 min, were maintained, as we obtained good performance at all concentrations of the standard curve (Fig. 2A).

**Table 2:** Recombinase polymerase amplification assay conditions for the detection of *Leishmania braziliensis*

Components	Initial concentration	Volume per reaction (µl)	Final concentration
TwistDx master mix	n/a	29.0	n/a
Nuclease-free water	n/a	6.6	n/a
LB-F	5 μM	4.8	410 nM
LB-R	5 μM	4.8	410 nM
LB-P	5 μM	0.6	50 nM
Endonuclease IV (Nfo)	10 units/µl	5.86	1 unit/µl
MgOAc	280 mM	2.5	11.83 mM
Template DNA	5 ng/µl to 0.5 pg/µl	5.0	0.4 ng/µl to 0.04 pg/µl

*n/a*: not applicable

**Table 3:** Recombinase polymerase amplification assay conditions for the detection of *Leishmania infantum*

Components	Initial concentration	Volume per reaction (µl)	Final concentration
TwistDx master mix	n/a	29.0	n/a
Betaine	0.8 M	6.6	0.09 M
LI-F	10 μM	4.8	810 nM
LI-R	10 μM	4.8	810 nM
LI-P	10 μM	0.6	100 nM
Endonuclease IV (Nfo)	10 units/µl	5.86	1 unit/µl
MgOAc	280 mM	2.5	11.83 mM
Template DNA	5 ng/µl to 0.5 pg/µl	5.0	0.4 ng/µl to 0.04 pg/µl

*n/a*: not applicable

In the RPA-LF assay for *L. infantum*, the primers and probe were used at 10 μM (the maximum concentration recommended by the manufacturer) (Table 3), to achieve better performance in terms of analytical sensitivity. To further optimize the visualization of the assay, we tested different incubation temperatures and times. The specific test line for *L. infantum* was observed at all temperatures and incubation times tested, but with the best resolution at 40 °C for 40 min (Fig. 2B). Due to the appearance of non-specific bands in the NTC, we added 0.8 M betaine solution to the RPA-LF reaction for *L. infantum*.

### Analytical sensitivity and specificity of RPA-LF assays

In the RPA-LF assay for *L. braziliensis*, an amplicon dually labeled with carboxyfluorescein and biotin was visualized as expected in the test line 2 of the lateral flow strip. The analytical sensitivity of the assay was 0.04 pg/µl. In the RPA-LF assay for *L. infantum*, an amplicon dually labeled with digoxigenin and biotin was visualized as expected in the test line 1. This assay had a detection limit of 0.04 ng/µl (Fig. 2B). The cross-test experiments did not reveal non-specific amplifications with the standardized assays; that is, the assays detected only the target species.

### Sensitivity, specificity, accuracy, positive and negative predictive values, and diagnostic agreement

**Fig. 3:**
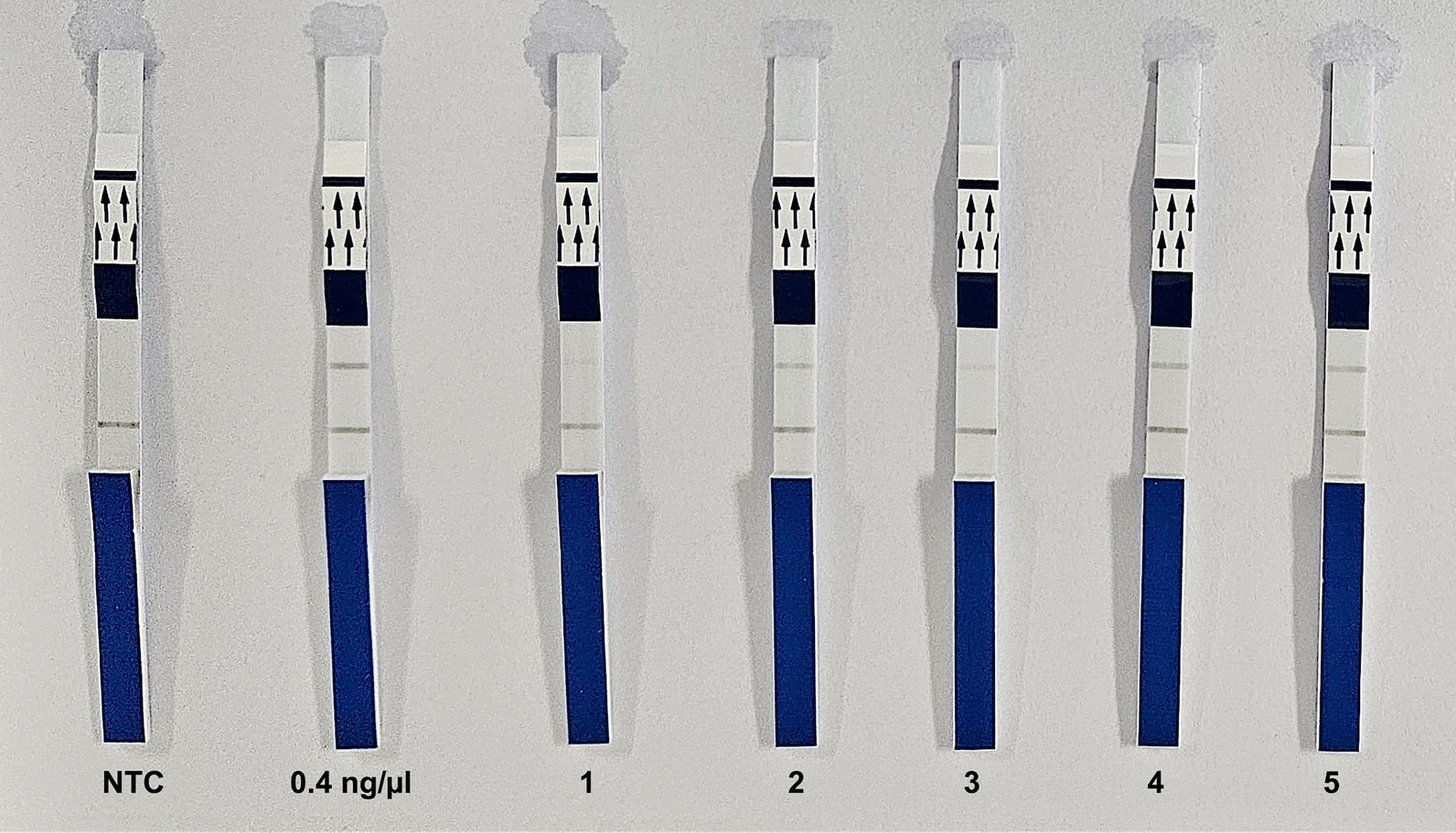
Results of the recombinase polymerase amplification lateral flow assay (RPA-LF) for *L. infantum* on DNA extracted from skin samples from naturally infected dogs (numbers 1–5)

The standardized *L. infantum* RPA-LF assay was tested with different types of dog samples (i.e., 34 skin and 36 bone marrow) from an area of active transmission in Brazil. A representative experiment is shown in Fig. 3.

The sensitivity, specificity, accuracy, and positive and negative predictive values of the standardized *L. infantum* RPA-LF assay in bone marrow and skin samples are shown in Tables 4 and 5. Overall, the *L. infantum* RPA-LF presented high sensitivity, specificity, and accuracy using skin samples. This assay presented a higher positive predictive value in skin and a higher negative predictive value in bone marrow (Table 4).

**Table 4:** Sensitivity, specificity, and predictive values of the recombinase polymerase amplification lateral flow assay (RPA-LF) for the detection of *Leishmania infantum* compared with conventional PCR (cPCR), using skin and bone marrow samples from dogs; all values are expressed as percentages (%)

Samples	*L. infantum* RPA-LF	cPCR		Se	Sp	Acu	PPV	NPV
		+	−					
Skin (*n* = 34)	+	23	0	92.0	100	94.1	100	81.8
	−	2	9					
Bone marrow (*n* = 36)	+	19	2	90.5	86.7	88.9	90.5	86.7
	−	2	13					

+ positive, − negative, *Se* sensitivity; *Sp* specificity, *Acu* accuracy, *PPV* positive predictive value, *NPV* negative predictive value

**Table 5:** Sensitivity, specificity and predictive values of the recombinase polymerase amplification lateral flow assay (RPA-LF) for the detection of *Leishmania infantum* compared with real-time PCR (qPCR), using skin and bone marrow samples from dogs; all values are expressed as percentages (%)

Samples	*L. infantum* RPA-LF	qPCR		Se	Sp	Acu	PPV	NPV
		+	−					
Skin (*n* = 34)	+	23	0	76.7	100	79.4	100	36.4
	−	7	4					
Bone marrow (*n* = 36)	+	21	0	70.0	100	75	100	40.0
	−	9	6					

+, positive, − negative, *Se* sensitivity, *Sp* specificity, *Acu* accuracy, *PPV* positive predictive value, *NPV* negative predictive value

The agreement between the *L. infantum* RPA-LF assay and the cPCR was almost perfect using skin samples and substantial using bone marrow samples (Table 6). The overall agreement with qPCR was moderate, but considering samples with *C*_q_ < 30 (moderate to high parasite load), the agreement was almost perfect (kappa = 0.807) (Table 7).

**Table 6:** Agreement between the recombinase polymerase amplification lateral flow assay (RPA-LF) for the detection of *Leishmania infantum* and conventional PCR (cPCR), using skin and bone marrow samples from dogs

Parameter	RPA-LF vs. cPCR, skin (*n* = 34)	RPA-LF vs. cPCR, bone marrow (*n* = 36)	RPA-LF vs. cPCR, all samples (*n* = 70)
Observed agreements	32 (94.1%)	32 (88.9%)	64 (91.4%)
Agreements expected by chance	19.8 (58.3%)	18.5 (51.4%)	37.8 (54.0%)
Kappa coefficient	0.859	0.771	0.813
Standard error of kappa	0.096	0.108	0.073
95% confidence interval	0.671–1.000	0.560–0.982	0.671–0.956
Classification	Almost perfect	Substantial	Almost perfect

**Table 7:** Agreement between the recombinase polymerase amplification lateral flow assay (RPA-LF) for the detection of *Leishmania infantum* and real-time PCR (qPCR), using skin and bone marrow samples from dogs

Parameter	RPA-LF vs. qPCR, skin (*n* = 34)	RPA-LF vs. qPCR, bone marrow (*n* = 36)	RPA-LF vs. qPCR, all samples (*n* = 70)	RPA-LF vs. qPCR, *C*_q_ < 30^a^ (*n* = 35)
Observed agreements	27 (79.4%)	27 (75.0%)	54 (77.1%)	32 (91.4%)
Agreements expected by chance	21.6 (63.5%)	20 (55.6%)	41.4 (59.2%)	19.4 (55.5%)
Kappa coefficient	0.436	0.437	0.440	0.807
Standard error of kappa	0.157	0.134	0.102	0.104
95% confidence interval	0.129–0.743	0.174–0.701	0.240–0.840	0.603–1.000
Classification	Moderate	Moderate	Moderate	Almost perfect

^a^In this analysis, all skin and bone marrow samples with a quantification cycle (*C*_q_) < 30 (moderate to high parasite load) and negative samples were included. Samples with a *C*_q_ > 30 were excluded

## Discussion

We successfully standardized two new RPA-LF assays for the detection of *L. braziliensis* and *L. infantum* in dogs. There was substantial agreement between the *L. infantum* RPA-LF assay and cPCR. Comparing to a highly sensitive qPCR assay [29], there was almost perfect agreement between the *L. infantum* RPA-LF and the qPCR results in samples with a *C*_q_ < 30. For samples with *C*_q_ > 30 (i.e., lower parasite load), the agreement was only moderate. This means that for dogs with a moderate to high parasite load, the RPA-LF would detect the parasite, whereas false negative results can occur in dogs with low parasite loads.

The superior analytical sensitivity of the qPCR as compared to RPA is acknowledged [20]. Therefore, the higher sensitivity of our qPCR as compared to the newly developed *L. infantum* RPA-LF assay was expected and may partly explain the moderate agreement between these assays, when also considering samples with a lower parasite load. Indeed, qPCR assays are known for their higher sensitivity, even compared to cPCR [28, 32]. In this regard, the *L. infantum* RPA-LF assay presented high sensitivity (> 90%) on both skin and bone marrow samples, using cPCR as a reference test. In particular, the *L. infantum* RPA-LF assay presented 92% sensitivity and 100% specificity on skin samples, with almost perfect agreement with cPCR. These data suggest that the *L. infantum* RPA-LF assay on skin samples could be an alternative for cPCR in remote areas lacking laboratory infrastructure for performing cPCR assays and gel electrophoresis.

In recent decades, molecular assays based on PCR have become more accessible and popular [2, 17]. PCR-based assays generally present good sensitivity in detecting *Leishmania* DNA in a wide range of samples, including from bone marrow, lymph node, skin, ocular swab, and even blood [2, 28, 29, 32]. However, molecular methods are still far from the reality of many veterinary practitioners working in areas where proper laboratory infrastructure is limited or lacking. Thus, the development of point-of-care molecular tools that could detect *Leishmania* DNA with good sensitivity and specificity would be a great advancement for the diagnosis of canine leishmaniasis in those areas.

In this sense, one of the main advantages of the RPA is its simplicity. The amplification process in RPA assays can occur at 30–42 °C [25, 33] and within 5–40 min [26, 34–38]. In fact, we obtained positive results at a wide range of temperatures and reaction times, with little variation in terms of band intensity. Again, this may be an important feature for an assay to be used under field conditions.

The present study has some limitations. Because of funding constraints, we tested a relatively limited number (*n* = 70) of samples from dogs. Similarly, we did not test the RPA-LF assay using samples from *L. braziliensis*-infected dogs. The next steps would be to test these assays on a larger number of samples from dogs infected by *L. infantum* and *L. braziliensis*. It would also be interesting to test the performance of the RPA-LF assay for *L. infantum* using less invasive samples, such as blood and conjunctival swab.

Finally, as RPA assays also have the potential to be applied for the point-of-care diagnosis of human leishmaniasis [20], this should be investigated using the assays developed in the present study.

## Conclusions

The RPA-LF assays developed herein may be promising molecular tools for the point-of-care diagnosis of *L. braziliensis* and *L. infantum* infections in dogs, particularly in remote areas lacking laboratory infrastructure. RPA-LF is a valid alternative to the more laborious and expensive qPCR and cPCR assays. Further research is needed to validate these assays on a larger number of samples and to confirm their applicability under field conditions.

## Data Availability

No datasets were generated or analyzed during the current study.
